# What Caused the Outbreak of COVID-19 in China: From the Perspective of Crisis Management

**DOI:** 10.3390/ijerph17093279

**Published:** 2020-05-08

**Authors:** Ziheng Shangguan, Mark Yaolin Wang, Wen Sun

**Affiliations:** 1 School of Public Administration, Hohai University, Nanjing 211100, China; sgzh@hhu.edu.cn; 2 School of Geography, the University of Melbourne, Melbourne, VIC 3010, Australia; 3 Asia Institute, the University of Melbourne, Melbourne, VIC 3010, Australia; 4 School of Criminal Law, East China University of Political Science and Law, Shanghai 200042, China

**Keywords:** COVID-19, public health, crisis management, big data, China

## Abstract

Since the first known case of a COVID-19 infected patient in Wuhan, China on 8 December 2019, COVID-19 has spread to more than 200 countries, causing a worldwide public health crisis. The existing literature fails to examine what caused this sudden outbreak from a crisis management perspective. This article attempts to fill this research gap through analysis of big data, officially released information and other social media sources to understand the root cause of the crisis as it relates to China’s current management system and public health policy. The article draws the following conclusions: firstly, strict government control over information was the main reason for the early silencing of media announcements, which directly caused most people to be unprepared and unaware of COVID-19. Secondly, a choice between addressing a virus with an unknown magnitude and nature, and mitigating known public panic during a politically and culturally sensitive time, lead to falsehood and concealment. Thirdly, the weak autonomous management power of local public health management departments is not conducive for providing a timely response to the crisis. Finally, the privatization of many state-owned hospitals led to the unavailability of public health medical resources to serve affected patients in the Wuhan and Hubei Province. This article suggests that China should adopt a Singaporean-style public health crisis information management system to ensure information disclosure and information symmetry and should use it to monitor public health crises in real time. In addition, the central government should adopt the territorial administration model of a public health crisis and increase investment in public health in China.

## 1. Introduction

Since the first known case of a COVID-19 infected patient in Wuhan, Hubei Province, China on 8 December 2019 [1], COVID-19 has spread to more than 200 countries and infected over three million people worldwide (as of 28 April 2020), causing a worldwide public health crisis. However, the COVID-19 pandemic has not only caused an unprecedented global health crisis, but has also triggered a worldwide economic downturn—“2020 is on track to witness the deepest global recession on a scale not seen since World War II” [2]. One of the major reasons for the current global public health crisis is the failure in virus control during its early stages in Wuhan and the remaining areas of Hubei province.

COVID-19 can result in infectious diseases of the respiratory tract which is similar to SARS [3], but is spreading much faster and wider than SARS. SARS broke out in Guangdong Province, China on November 16 2002, and spread to 24 of a total of 34 provinces, municipalities and autonomous regions in China in six months [4], while COVID-19 spread from a single city to the entire country (all 34 provinces, municipalities and autonomous regions) in just 54 days. From the typology of crises proposed by Gundel [5], it is easy to attribute the public health crisis caused by COVID-19 to an unexpected crisis, which means the crisis is hard to predict but easy to influence. However, China failed to influence the rapid spread of COVID-19 even though it had gained valuable experience in dealing with a public health crisis after SARS and later established a disease control and prevention system led by the Center for Disease Control & Prevention (CDCP) [6]. This is a question worthy of reflection.

The existing literature on COVID-19 focuses on pathological characteristics of patients [7,8], gene sequencing of COVID-19 [9,10], demographic features of the patients and fatality rate [11,12,13]. Such research is largely based on clinical diagnosis, general epidemiological research, response tactics (isolation), social distancing and community containment. What is missing is the mismanagement factors contributing to the sheer speed of spatial expansion and dramatic increase in infected people. This article attempts to fill this research gap through analysis of big data, officially released information and other social media sources to understand the root cause of the crisis from the aspects of China’s current management system and public health policy. To do so, this paper draws on structural–institutional perspectives to examine the coordination structure and mechanisms in the COVID-19 crisis management system.

## 2. Crisis Management Literature

Crisis management involves multiple disciplines [14] including psychology, sociology, political science and management science [15]. The analytical methods of systemic approaches [16] and resilience engineering [17,18] are usually adopted in crisis management. Rasmussen adopted systemic approaches to build a crisis management system of socio-technical which is divided into three levels from top to bottom, namely: Government, Regulators and Associations and Company. They also explained the structure, goals, constraints, pressures and operating limits of the system [19]. Based on resilience engineering, Hollnagel et al. pointed out that a system that can resist a crisis requires four capabilities: (1) its ability to anticipate the occurrence of a perturbation; (2) its ability to monitor its operating condition to maintain control of its operations (during the perturbation); (3) its ability to respond when the disturbance is there; (4) its ability to learn from the occurrence of a perturbation [20]. Thus, it can be seen that crisis management involves multiple subjects and they work together to prepare for, handle and recover from the crisis. In public health crisis management, five factors are usually considered [21,22,23]: (1) information disclosure or control; (2) assessment of dangers and threats; (3) establishment of crisis information communication channels and health education platforms; (4) the making of and implementation of strategic crisis response plans; (5) overall mobilization of critical resources.

The first factor relates to crisis information disclosure and control, which is a choice for the authority to take. Such a choice is viewed as having a direct impact on people’s emotional reactions towards the crisis and thus is a foundation of crisis management. When an unknown crisis suddenly breaks out, people display negative psychological characteristics such as high anxiety, tension, depression, hostility, guilt and shame [24]. If these negative emotions are not well controlled, it will greatly hinder the effective implementation of crisis management [25]. Rosenthal pointed out that the government usually has two choices in the management of people’s emotions: controlling information and disclosing information. Controlling information requires minimizing external participation, media attention, and public initiatives. When disclosing information, the government takes the opposite position and actively encourages the mobilization of various forces. Information disclosure has obvious advantages, but the reality is that some governments take an information control approach to deal with a crisis [26]. Perry’s work shows that when a crisis cannot be quelled in the short term, controlling information normally causes even greater panic [27].

The second factor relates to scientific and effective assessment of the crisis which is performed for the later formulation of intervention policies [28,29]. Such crisis assessment should be based on accurate information because information deviation will directly lead to the expansion of the crisis [30]. Therefore, in the early stages of the crisis, investigations should be carried out quickly to collect information and carefully verify the accuracy of all information. Meanwhile, real-time monitoring of the crisis should be conducted to ensure the timeliness of information [31]. For a general epidemic, after collecting a large amount of data, experts and scholars will assess its infectivity, susceptible population and mortality [1]. Some scholars also use mathematical models to estimate the number of people infected by the epidemic [32]. COVID-19 has also been studied in mathematical modelling [33]. With the development of science and technology, the methods of crisis assessment have thus become diversified [21].

The third factor is establishment of crisis information communication channels and health education platforms, which is an important communication component in crisis management. Their functions include the release of risk information, evacuation notices, risk prevention measures and available assistance by public institutions [34]. Because different types of crises present different forms of threat, the emphasis on communication and education is different [35]. In a public health crisis, relevant agencies need to provide the public with information on the source of the disease, preventative measures, symptoms of the disease, treatment methods and routes of transmission in a timely manner [36]. Through communication channels and health education platforms, the public can respond to the crisis autonomously, thereby helping the government control the crisis [37].

The fourth factor is the making and implementation of strategic crisis response plans, and this factor is crucial for the government [38]. Theoretically, government departments can effectively curb the crisis by formulating corresponding policies based on scientific crisis assessment [39], but for the government, the key of crisis control in crisis management is to gain time [40]. Rosenthal et al. believe that the longer it takes for government decision-making to take place, the more political criticism and rumors may be induced [41], and these factors often cause the collapse of the response policies [32]. Therefore, government departments must intervene immediately when the crisis comes to light. Delaying every second will negatively impact the government; however, it must take sufficient time to formulate efficient and reasonable policies. This presents a dilemma for the government.

The fifth factor is overall mobilization of critical resources. During the Bird Flu and SARS in China, resource allocation was far beyond the capacity of local resource management, causing shortages of medical substances and food supply [42]. Timely and effective allocation and transportation of emergency resources can reduce losses caused by public crises [43]. As time, quantity and quality of the resources are the key limitation factors, emergency managers do have to find an optimal schedule for assigning resources to the affected areas [44]. A reasonable public health crisis response system should guarantee a certain reserve of medical space, medical materials and medical staff, which is also a prerequisite for resource mobilization [45].

Based on the five factors of crisis management highlighted through a literature review, this paper intends to explain the problems in China’s handling of the public health crisis caused by COVID-19 and discusses the causes of such problems.

## 3. Approaching the Root Cause

### 3.1. Data Collection

All research data for this paper comes from several sources, including Chinese government official reports and news, big data statistics, official statistical data and international journals and the media.

(1) Chinese Government Official Reports and News

Chinese government official reports and news were mainly sourced from Caijing (http://www.caixin.com/2020-01-20/101506242.html) and Nanfang Metropolis Daily (https://m.mp.oeeee.com/h5/pages/v20/nCovTimeline/?from = groupmessage&isappinstalled = 0), which are used to obtain the latest information and reports on COVID-19.

(2) Big Data Statistics

The big data statistics platform uses Baidu (similar to Google) can provide a media index (http://index.baidu.com/v2/index.html#/) of certain keywords reported by the media and emigration index (https://qianxi.baidu.com/). 

The media index reflects the number of keywords related to the news reported by the media and included in the Baidu news channel. The keywords searched in this paper were “Wuhan pneumonia” and “novel coronavirus,” because the Chinese media universally reported COVID-19 using these words as the key title words. 

The emigration index reflects the scale of emigration and is the normalized value. In this paper, we searched the emigration index of Wuhan and compared this index against the 25 days pre-Chinese New Year in 2020 and 2019: two periods of data comparison—1 January 2020 to 25 January 2020 (Chinese New Year was 25 January 2020) and 12 January 2019 to 5 February 2019—when there was no COVID-19 (Chinese New Year for 2019 was 5 February 2019).

(3) Official Statistical Data and International Journals and Media

Our official statistical data was collected from relevant Chinese departments, international journals and the media. China’s official sources come from the official website of national CDCP (http://www.nhc.gov.cn/), the national Health Commission (HC, http://www.nhc.gov.cn/), and the national Bureau of Statistics (BS, http://www.stats.gov.cn/). Our international journal references mainly cite publications by the *Lancet*, which covers the largest volume of COVID-19 papers. Data from these departments and journal articles were used to obtain the number of people infected with COVID-19, infection characteristics, control information and public health expenditure.

### 3.2. A Crisis Dividing Line

Tracing COVID-19 development in China, we can see that 25 January 2020 is a dividing line. Our study concentrates our research attention to the period from 8 December 2019 (the first known case) to 25 January 2020 when the central government commenced its tough lockdown policy. This was a stage of confusion and chaos. Initially it was called Wuhan pneumonia (“Wuhan fei yan” in Chinese pinyin). On the morning of 26 December 2019, Mr. Jixian Zhang, the director of respiratory medicine of Hubei Provincial Hospital of Integrated Chinese and Western Medicine (Wuhan) found four abnormal cases of pneumonia and he reported this to Wuhan CDCP the next day. On 28 and 29 December 2019, the outpatient department of that hospital admitted another three patients from the Wuhan South China Seafood Market. The symptoms of these seven patients were similar. On 5 January 2020, the Wuhan HC confirmed that there were 59 patients with an unexplained pneumonia diagnosed in Wuhan. On 9 January 2020, Chinese authorities officially confirmed the novel coronavirus as the pathogen of “Wuhan pneumonia.” Since then, Chinese media has described “Wuhan pneumonia” as a new coronavirus (later named COVID-19 by the World Health Organization). On 11 January 2020, the Wuhan HC advised that there were 41 people infected with COVID-19 and the number of infected people increased by 4, 17, and 59 on 16, 17 and 18 January, respectively. Since 19 January 2020, new COVID-19 infections occurred in other provinces, municipalities and autonomous regions in China. By then, a large-scale outbreak of COVID-19 occurred in China. On 23 January 2020, the Wuhan Municipal Government suspended city buses, subways, ferries, long-distance passenger transportation, airports and train stations in the city, prohibiting citizens from leaving Wuhan. With the rapid spread of the virus in China, every level of government successively initiated first-level responses to major public health emergencies (the highest level of public health incident response) on 23–25 January 2020. On 25 January 2020, China officially entered the national epidemic control stage. Thus, the problems in crisis management for China mainly occurred prior to 25 January 2020.

## 4. Problems in COVID-19 Crisis Management

This paper reports our key findings within the following five factors.

### 4.1. Factor 1. Information Disclosure or Control: Early Media Controls Left Most People Defenseless against COVID-19

**Figure 1:**
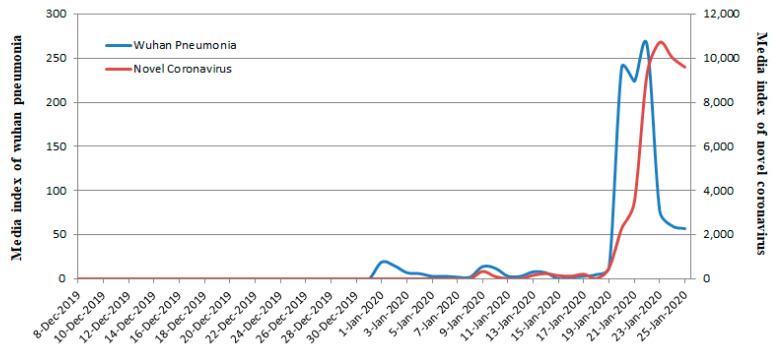
Frequency of the media keywords “Wuhan pneumonia” and “Novel Coronavirus” from 8 December 2019 to 25 January 2020.

Research concludes that information control can temporarily prevent the spread of rumors in the crisis and eliminate the fear of the public [25]. However, the public experience greater fear if the crisis erupts in a large scale, so this is generally not advocated in crisis management [46]. The Chinese case confirms that information control deprives people of their ability to prevent and resist crises. In fact, in the early stages of COVID-19, the Chinese government adopted information blockades and controls to prevent public panic, which resulted in most people being unprepared for COVID-19. We searched Baidu for the keywords “Wuhan pneumonia” and there was no single media report on “Wuhan pneumonia” prior to 31 December 2019 (Figure 1). There were very few reports during the period 1 January 2020 to 19 January 2020. Our search of the media keyword “Novel Coronavirus” found no single report on the novel coronavirus prior to 8 January 2020. 20 and 21 January 2020 witnessed a large search for “Wuhan Pneumonia,” which was replaced by “Novel Coronavirus” after 23 January 2020. In summary, the Chinese government imposed a strict media blockade on the report of COVID-19 before 31 December 2019 and controlled it from 1 January 2020 to 19 January 2020. It was not until 20 January 2020 that the Chinese media began to cover it extensively.

Due to the media blockade and control of information about COVID-19, the Baibuting Community in Wuhan held a banquet with more than 40,000 families on 18 January 2020, which was approved to be the single most serious infectious event in Wuhan. Knowing nothing about COVID-19, people in Wuhan still visited population-intensive places such as shopping malls, supermarkets and entertainment places in large numbers until 20 January 2020. This accelerated the rapid spread of COVID-19. A few medical workers did try to warn of the outbreak, but all were silenced, including the famous “Coronavirus Whistleblower” Dr Li Wenliang, who tried to warn his medical university classmates in his WeChat circle of the outbreak, but was accused of spreading fake information by local police [47], although afterwards, the Chinese government acknowledged Dr Li ’s contribution and recognised him as a martyr. In summary, the control of information and media silence directly resulted in most people being unprepared during the COVID-19 outbreak.

### 4.2. Factor 2. Assessment of Dangers and Threats: Early Concealment of the Epidemic Caused an Error in Chinese HC’s Assessment of the Crisis

In a public health crisis, timely and effective access to information is critical. However, the HC in each province, municipality and autonomous region had concealed the epidemic in the early stages of COVID-19, which caused the national HC to make an incorrect assessment of the reality of the situation. 

**Figure 2:**
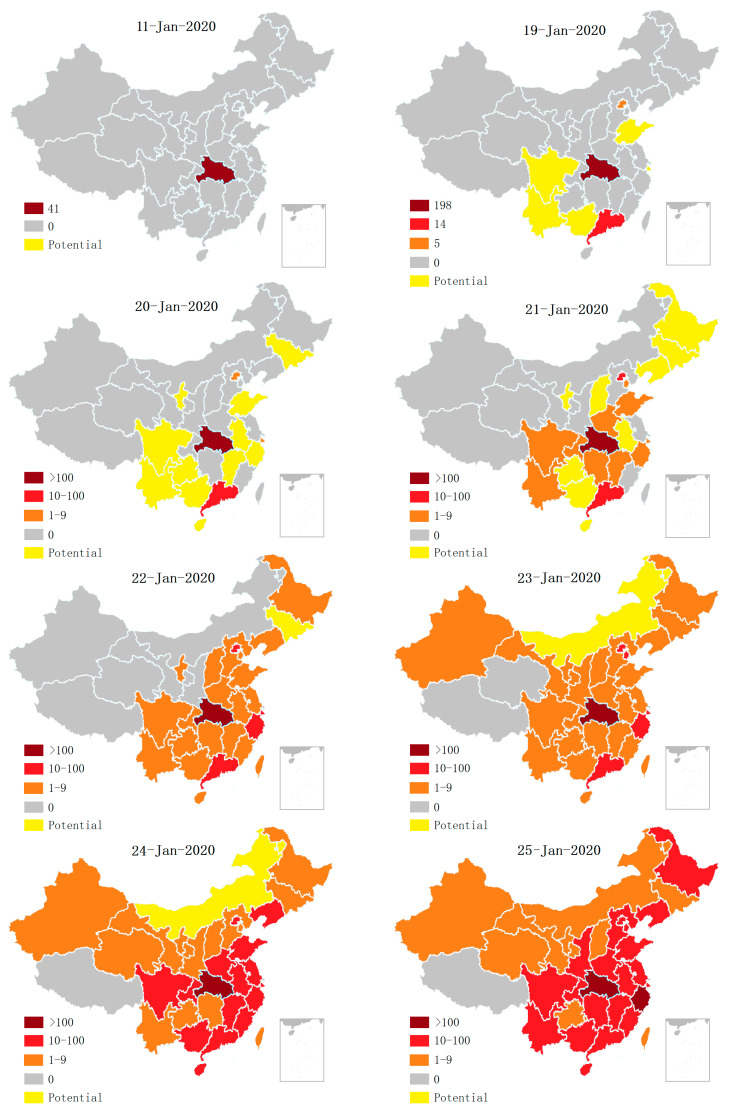
Spatiotemporal distribution of COVID-19-infected patients.

Figure 2 shows that the period from 20 January to 25 January 2020 registered the fastest spread of COVID-19, whilst the official record shows no increase in the number of infected cases in Hubei Province from 11 January to 15 January 2020 with no emergency response measures applied, indicating that Hubei HC had concealed the epidemic. Some scholars have questioned the under-reporting of infected people by China [48]. By constructing a mathematical model, Imai et al. concluded that as of 12 January 2020, there should have been 1723 COVID-19-infected patients (not the officially reported 41 patients in Wuhan) [49]. Wu et al. calculated that as of 25 January 2020, there had been 75,815 COVID-19 infected patients in Wuhan using the data of patients given by national HC from 31 December 2019 to 28 January 2019 [50].

Under-reporting of Chinese COVID-19 cases can be contrasted to the experience of China’s neighboring countries. As revealed in Section 3.1, before 19 January 2020, COVID-19 did not spread on a large scale in China, but only spread within the Hubei Province. This was impossible as there was one case of a COVID-19 patient in Thailand reported on 13 January 2020 and another case in Japan on 16 January 2020. Wuhan’s connection to these two countries is far less frequent than other Chinese provinces. How could there be no spread within China when it had spread to Thailand and Japan? In addition, according to spatiotemporal distribution of people infected with COVID-19 as shown in Figure 2, Guangzhou, Shanghai and Beijing, which are further away from Hubei Province, had COVID-19 cases earlier than other provinces and municipalities around Hubei Province. However, according to a Baidu search of Wuhan’s population emigration index, before 25 January about 400,000 people left Wuhan moving to Guangdong province, 120,000 to Shanghai, 120,000 to Beijing, and 680,000 to the provinces and municipalities around Hubei province. This showed that the population who moved to the neighboring provinces and municipalities is almost equal to that of Guangzhou, Shanghai and Beijing, so it is impossible for the provinces and municipalities around Hubei to have had no infectious cases to report. This suggests that the HC of each province, municipality and autonomous region outside Hubei province had initially concealed the epidemic.

### 4.3. Factor 3. Establishment of Crisis Information Communication Channels and Health Education Platforms: Officials Denying Interpersonal Transmissions

In a public health crisis caused by infectious diseases, public health agencies should establish communication channels and health education platforms in a timely manner and communicate the source, symptoms, transmission mode of the disease and isolation measures to the public [34]. However, the national CDCP did not fare well in the early debate about whether COVID-19 can be transmitted from person to person. The explanation from the national CDCP about whether COVID-19 can be transmitted from person to person is shown in Table 1 below.

Table 1 shows that the national CDCP did not explicitly point out that COVID-19 could be transmitted from person to person before 19 January 2020. It was not until 20 January 2020 that Nanshan Zhong, an academic of the Chinese academy of engineering, confirmed that COVID-19 could be passed from person to person. The official media did not report this before 20 January 2020 despite cases of medical staff being infected by person-to-person transmission. Interesting enough, authors including staff working in China’s national CDCP and Wuhan’s CDCP published a paper in an English academic journal—the *New England Journal of Medicine*—with a conclusion that interpersonal transmissions had occurred among close contacts since the middle of December 2019 [7]. Another English paper published in *The Lancet*, whose leading author Chen Wang is an academic of the Chinese Academy of Sciences, also mentioned 16 cases of medical workers infected with COVID-19 in December 2019 [8]. Another English paper published in *The Lancet* by Chen described in detail a case of family cluster transmission and demonstrated that COVID-19 can be transmitted from person to person. The patients mentioned in the article were admitted to the hospital on 10 and 11 January 2020 [51]. The fact that seven medical staff were infected between 1 January 2020 to 11 January 2020, with eight more infected between 12–22 January 2020 was released to the public by various Chinese media sources after the central government officially accepted interpersonal transmission [52].

### 4.4. Factor 4. Making and Implementation of Strategic Crisis Response Plans: Wuhan Government’s Slow Response to the Crisis Leading to 5 Million People leaving Wuhan

Generally speaking, the government plays an important role in crisis management, and it can respond to the crisis efficiently by formulating a unified response policy [46]. If the government fails to formulate a response in time, it will cause the public to panic and lose confidence in the government [40]. In the process of the crisis management of COVID-19, the Wuhan municipal government could have taken preventative measures at a much earlier time. Based on various media reports, Table 2 summarizes the key time points of other countries in taking preventative measures compared to the Wuhan government who did not take measures at such times. As early as 1 January 2020, hospital doctors in Wuhan found an unknown type of pneumonia based on medical testing reports (later it was confirmed that the nucleotide had the homology of COVID-19, which is 82% similar to the SARS virus [53]). However, instead of strengthening the protective measures against COVID-19, the Wuhan local government accused the doctors of spreading rumors. At the same time, Singapore and Hong Kong had already introduced quarantine measures on 3 and 4 January 2020, respectively. After 13 January 2020, COVID-19 infected patients appeared in countries other than China, indicating that COVID-19 had spread widely. However, the Wuhan government did not introduce the lockdown policy until 23 January 2020.

**Figure 3:**
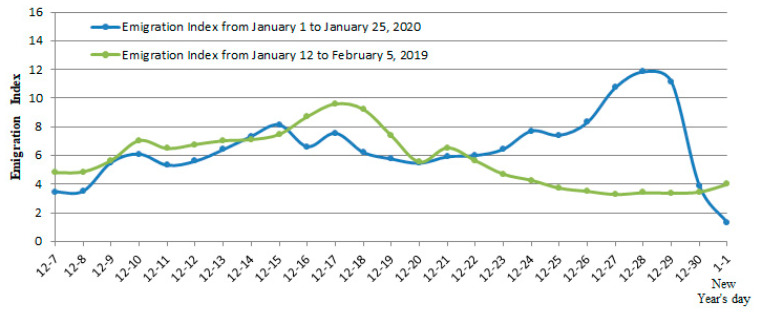
Emigration index in 2020 and 2019.

Another fundamental mistake that the Wuhan government made was that it allowed five million people to leave Wuhan before China quarantined the city [54]. Although many left due to the Chinese New Year, others also left to avoid quarantine. This is supported by our data comparison. Using the Chinese lunar calendar time table to compare to Wuhan’s emigration index (the emigration index here is the normalized value) from 1–23 January 2020 and the emigration index from January 2019 to 5 February 2019, we found that the emigration index in 2020 and 2019 is basically the same before lunar 22 December 2019 and shows the opposite trend from lunar 23 December 2019 to the Chinese New Year (see Figure 3). Normally, the closer to Chinese New Year, the fewer people move out of Wuhan, but the trend shows that the opposite occurred in 2020, indicating that many Wuhan residents had been aware of the crisis and started to take the initiative to leave. 

### 4.5. Factor 5. Overall Mobilization of Critical Resources: Insufficient Medical Resources to Respond to the Public Health Crisis Accelerated the Spread of COVID-19

The final part of this paper investigates the availability of public health crisis resources. Public health crisis causes a surge in demand, especially for medical resources in the short term, so the government needs to coordinate the mobilization of resources [44]. Starting from lockdown, under the leadership of the central government the Chinese governments at all levels mobilized support to Wuhan including transporting a large number of medical supplies and sending nearly 29,445 medical staff (excluding military medical staff) to Wuhan. This also included building a “Huosheng” mountain hospital (with 1000 beds) and a “Leisheng” mountain hospital (with 1600 beds) in Wuhan within 10 to 11 days. However, all these achievements do not overshadow the problem of a serious shortage of infectious disease medical facilities and materials in China. China is not ready to cope with a large-scale infectious disease.

For a long time, China has had insufficient reserves of medical resources for infectious diseases. Take Wuhan as an example: the epidemic started and 14 million people (8.5 million local residents and over 5 million migrant workers or students whose hometowns are in other parts of China) were able to access only two hospitals which treat infectious diseases. The total number of beds in these hospitals is around 900 which equates to 0.64 beds per 10,000 people. This is far lower than the standard for the number of beds in infectious disease hospitals in China of 1.2 to 1.5 beds per 10,000 people [52]. On 23 January 2020, Wuhan had no hospital beds available for infected people and those who suspected they were infected, so the “Huosheng” mountain hospital was built, followed by the “Leisheng” mountain hospital on 25 January 2020. Because of the shortage of beds, doctors could only advise that patients who suspected they had COVID-19 and those with mild symptoms be isolated at home. This led to a sharp increase in the number of family cluster transmissions [55]. In addition, the shortage of masks, goggles and protective clothing also caused a large number of medical staff to be infected in the early stages. According to the “Analysis of Epidemiological Features of New Coronavirus Pneumonia” issued by the national CDCP, it is known that by 17 February 2020, a total of 3019 medical staff were infected with COVID-19.

## 5. Discussion

This section will first identify what the problems in China’s public health crisis management are, and then explain the key causes.

### 5.1. The Major Problems in COVID-19 Crisis Management

(1) The Media Lost Its Supervisory Function

Chinese media are directly managed by the Chinese government, which leads them to serve the government. After the government monopolizes and controls the information resources, it can be selective in reporting and releasing information. This results in information submission errors [56], which directly promotes the public’s failure to obtain real and effective information in a timely manner. In this case, the Chinese media did not help the government observe and warn of the public crisis, nor did it monitor the government’s deficiencies in handling public crises.

(2) A choice between addressing a virus with an unknown magnitude and nature, and mitigating known public panic during a politically and culturally sensitive time

Stage 1 was both politically and culturally sensitive given that nobody wanted to create any unnecessary public panic during the period leading up to Chinese New Year with a virus that was still relatively unknown in terms of magnitude and nature. Wuhan and the rest of the local governments were getting ready in early January for the most important local yearly political meetings—the so-called “two sessions” (the National People’s Congress and the Chinese People’s Political Consultative Conference (CPPCC)). During the “two sessions,” the top priority is to maintain stability and keep this period free from problems. This is also why when experts from Beijing arrived to investigate the atypical pneumonia outbreak, Wuhan officials tried to avoid two things: upsetting Beijing and causing a public panic in advance of these two important meetings. Therefore, they took a multipronged approach to control information about the outbreak [57] Local officials concealed the facts due to the nature of the political climate and timing of when this was happening.

(3) Weak Autonomous Management Power of Local Public Health Management Departments

China’s public health management system does not allow local departments to declare infectious disease crises even in their own jurisdiction. It is the State Council which possesses the power to declare a crisis involving a statutory infectious disease and to draft and implement contingency plans. Meanwhile, local departments are asked to take appropriate measures to prevent diseases according to the epidemic prevention level set by the State Council for Infectious Diseases. Thus, in Wuhan’s case, Wuhan HC and Wuhan CDCP had no power to declare an infectious disease crisis or take any initial measures before authorization. More and more scholars believe that power concentration is not conducive to timely response to crises and multi-centralization of crisis management has become a trend [26]. It is important for the local departments to successfully combat any infectious disease because it obtains the information fast and should have the discretion to manage it to prevent a large-scale outbreak [40].

(4) Privatization of Public Hospitals Leads to Insufficient Public Health Medical Resources

**Figure 4:**
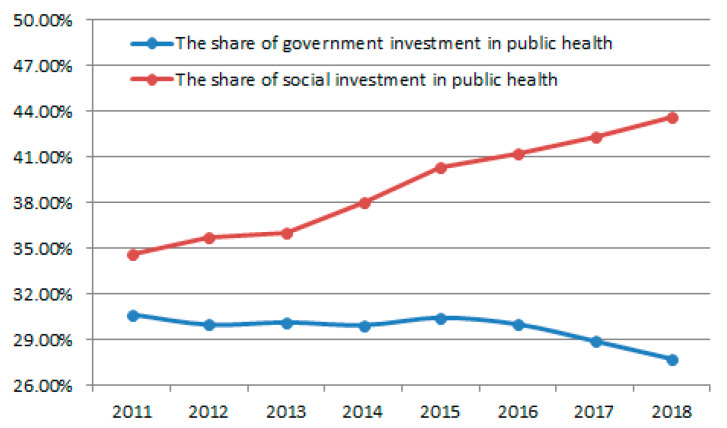
The share of government investment and social investment in public health from 2011 to 2018.

A significant healthcare trend in China is the increasing privatization of medical hospitals and clinics based on a belief that private hospitals are more efficient [58]. Up to 2020, the number of private hospitals in China account for over 66% of all Chinese hospitals [59]. The privatization of public hospitals has attracted a large amount of social capital, effectively reducing government expenditure on health. As can be seen from Figure 4, the share of government investment decreased from 30.66% in 2011 to 27.74% in 2018, and the share of social investment increased from 34.57% in 2011 to 43.66% in 2018. However, the privatization of public hospitals has resulted in insufficient public health and medical resources. On the one hand, due to the low frequency of public health disease outbreaks, private hospitals have invested less in public health care based on market demand. On the other hand, due to the decrease in government investment in public health, a large number of technical personnel in public health have been drained, resulting in a serious shortage of public health staff [52].

### 5.2. What Is Wrong with China’s Public Health Management Systems?

Based on Rasmussen’s socio-technical management system [19], we can frame China’s public health management systems into a three tiered hierarchical system. The Chinese government is at the top level and they are both the makers and implementers of the law. The regulators and associations, such as HC and CDCP, respectively, are at the second level. They develop crisis management norms based on the law and oversee the implementation. At the very bottom level are the public who selectively cooperate with the above two levels of management based on personal interests. During the COVID-19 crisis, the Chinese public was generally considered to be cooperative with the government and relevant health management departments [60], the public has little disturbance to the public health management system.

**Figure 5:**
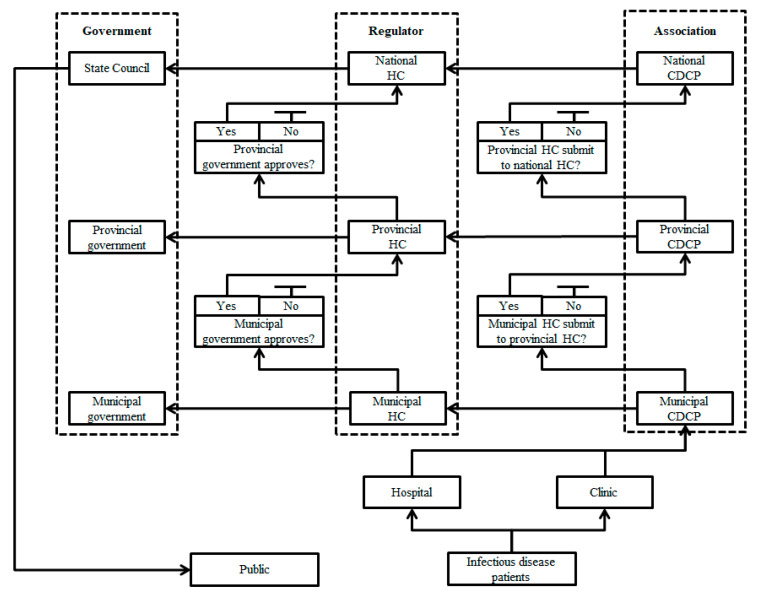
Information transmission path of China’s public health management system.

Using this framework, we have summarized the information flow path of China’s public health management system in Figure 5. This figure shows the many checkpoints required for an infectious disease case to reach the public. We can use this to explain what caused the delay and ignorance of releasing early COVID-19 information. After receiving confirmed cases from Wuhan’s hospital or clinic, the COVID-19 infectious disease information followed two separate paths. Wuhan Municipal CDCP reported its first COVID-19 case to Wuhan Municipal HC on December 8, 2019, but it could not report the same information to Hubei Provincial CDCP until after Wuhan HC had submitted COVID-19 information to Hubei provincial HC. A similar prerequisite applied to Hubei provincial CDCP which reported to National CDCP after Hubei provincial HC reported to national HC. The most critical requirement for smooth information transmission was that any local HC could not pass COVID-19 information to another upper level HC without permission from its local government. Therefore, Wuhan HC could report to Hubei provincial HC only if Wuhan municipal government approved it to do so. Similar approval was required from the Hubei provincial government as Hubei provincial HC reported to national HC on 31 December 2019. The final stage is that State Council released the COVID-19 infectious disease information to the public after it received the confirmed information from the national HC on 11 January 2020. We can see that it took a total of 34 days from the first confirmed COVID-19 patient reported by the Wuhan Municipal CDCP to the congress to the release of COVID-19 information to the public. We can also see, as the most professional of all the disease management associations, Wuhan municipal CDCP basically did not have any real authority to transmit disease information. It could neither issue an early warning of infectious diseases to the public nor take any emergency measures. The only authority able to release the early warning is the State Council. The lack of independent management power by the local CDCPs directly led to a serious lag in the release of disease information and the implementation of emergency measures.

## 6. Conclusions

China’s COVID-19 crisis management can be roughly divided into two stages. Since 25 January 2020, the Chinese central government launched an unprecedented national campaign to contain the disease. Now, China is winning the battle and the rest of the world is fighting. However, China has paid a huge price for its initial delays and slow response.

Strict government control over information was the main reason for the early media silencing, which directly caused most people to be unprepared for COVID-19. In fact, the same problem occurred during the SARS outbreak [52]. We can see that Chinese local government officials seem to be habitually choosing to underreport bad news for fear of economic losses or criticism from upper level officials, which would impact their personal political achievements. 

The weak autonomous management power of local governments is not conducive to taking a timely response to the crisis. Critical decisions about combating COVID-19 must be made and implemented under considerable time pressure. Local authorities need to take action quickly. Finally, the lack of appropriate medical assistance given by private hospitals to help manage COVID-19 should inform Chinese and relevant authorities to rethink China’s health system and reform and invest more in its public health network.

Ultimately, this paper covers two broad dimensions of COVID-19 crisis management: technical/physical and political. Our findings further confirm that crisis management involves multiple subjects and they have to work together to prepare for, handle and recover from crisis. The five commonly considered factors in public health crisis management are a useful framework to analyze the problems and major causes of the COVID-19 crisis in China. This crisis management analysis framework could be used to examine why China’s powerful national campaign has successfully contained COVID-19 in stage two and more interestingly, how some of the lessons learned from stage one had been immediately overcome in stage two. Each of the five factors has been addressed differently compared with stage one. For example, the central government’s top-down system was able to mobilize nearly all relevant organizations and services to support the lockdown of the whole nation (Factor 5); the public has been well informed: the government and media have provided continuous and clear COVID-19 information to the public (Factor 1); professional infectious disease control experts assess the virus threat regularly (Factor 2); COVID-19 public health education platforms are free for people to access (Factor 3); and the officials’ top priority on their daily agenda at every level of government is to make the right decisions at the right time—officials carefully listen to health experts in managing the local response action plan (Factor 4). More importantly, the world could learn more from China’s response to the COVID-19 outbreak.

Late intervention to COVID-19 is not an issue unique to China. In fact, countries like the United States and many European countries have also experienced some lag in the process of handling the crisis despite observing the Chinese demonstrating the serious consequences of delaying any response during stage one. Assigning blame is unproductive to the conversation, rather, this paper seeks the answer how China can or should make structural and management adjustments to its process of intervention so as to help the management of the future crises. Therefore, it is time to consider the following policy recommendations:

First, in the current organizational management structure in China, the main reasons for media loss and politics overruling the truth are information control and information monopoly. Singapore’s public health management system provides an idea for China’s future information management. Singapore has established a case management system (CMS) which collects information on hospitals, Ministry of Health, Ministries of Education and general practitioners via the Internet. The system can identify suspected and confirmed patients through the information collected and provide early warning flags. It also closely monitors close contacts of suspected and confirmed patients and makes available real-time information on patients, suspected cases and potential cases. China should adopt a similar system to make the epidemic information public.

Secondly, introducing a local territorial management model should be explored. The local public health management departments do not have autonomy to take timely action. China should adopt a local territorial management model such that as soon as a crisis occurs, local public health management departments can immediately release information to the public and take prompt measures according to the epidemic situation. Any initial delay will result in large damage down the track.

Finally, increasing government public health investment is key. At present, China’s CDCP is severely short of public health technical personnel and hospitals are short of public health medical resources. Therefore, the Chinese government should increase public health investments in training professional technicians and increasing medical resource reserves. To increase training, further investment in relevant education is required. To boost medical resource reserves, a medical emergency supply network system could be constructed [61]. This system could cover all cities and be funded by local governments in major infectious disease hospitals and general hospitals. Each hospital should have adequate emergency wards and medical supplies so that if an infectious disease were to arise, each type of hospital could cope with treating patients.

## Figures and Tables

**Table 1:** The official explanation by the national Center for Disease Control & Prevention (CDCP) about the transmission route of COVID-19.

Time	Official Explanations
21/12/2019	No obvious human-to-human transmission found; no medical staff found to be infected
5/01/2020	No clear sign of human-to-human transmission found; no medical staff found to be infected
10/01/2020	No medical staff found to be infected
11/01/2020	No clear evidence of human-to-human transmission found
14/01/2020	Limited human-to-human transmission is not excluded
16/01/2020	No clear evidence of human-to-human transmission found, the possibility of human-to-human transmission cannot be ruled out, but the risk of sustained human-to-human transmission is low
19/01/2020	The transmission route is not yet fully understood
20/01/2020	COVID-19 can transmit from person to person

Source: Chinese authoritative media.

**Table 2:** Important time points to take preventative measures.

Time	Events
1/01/2020	Eight medical doctors in Wuhan were summoned by local public security organs after calling out the unexplained pneumonia SARS on the WeChat platform based on medical testing reports, including Dr. Wenliang Li, known as the Chinese whistleblower
3/01/2020	Singapore quarantined passengers on flights from Wuhan
4/01/2020	Hong Kong government launched “serious” emergency response level for public health.
13/01/2020	First COVID-19 patient in Thailand
16/01/2020	First COVID-19 patient in Japan
17/01/2020	USA quarantined passengers on flights from Wuhan
20/01/2020	First COVID-19 patient in Korea
22/01/2020	First COVID-19 patient in America

Source: Chinese authoritative media.
